# Modulating the global orientation bias of the visual system changes population receptive field elongations

**DOI:** 10.1002/hbm.24909

**Published:** 2019-12-24

**Authors:** Christian Merkel, Jens‐Max Hopf, Mircea Ariel Schoenfeld

**Affiliations:** ^1^ Department of Neurology Otto‐von‐Guericke University Magdeburg Germany; ^2^ Department of Behavioral Neurology Leibniz Institute for Neurobiology Magdeburg Germany; ^3^ Kliniken Schmieder Heidelberg Germany

**Keywords:** functional MRI, Orientation adapter, Population receptive fields

## Abstract

The topographical structure of the visual system in individual subjects can be visualized using fMRI. Recently, a radial bias for the long axis of population receptive fields (pRF) has been shown using fMRI. It has been theorized that the elongation of receptive fields pointing toward the fovea results from horizontal local connections bundling orientation selective units mostly parallel to their polar position within the visual field. In order to investigate whether there is a causal relationship between orientation selectivity and pRF elongation the current study employed a global orientation adapter to modulate the orientation bias for the visual system while measuring spatial pRF characteristics. The hypothesis was that the orientation tuning change of neural populations would alter pRF elongations toward the fovea particularly at axial positions parallel and orthogonal to the affected orientation. The results indeed show a different amount of elongation of pRF units and their orientation at parallel and orthogonal axial positions relative to the adapter orientation. Within the lower left hemifield, pRF radial bias and elongation showed an increase during adaptation to a 135° grating while both parameters decreased during the presentation of a 45° adapter stimulus. The lower right visual field showed the reverse pattern. No modulation of the pRF topographies were observed in the upper visual field probably due to a vertical visual field asymmetry of sensitivity toward the low contrast spatial frequency pattern of the adapter stimulus. These data suggest a direct relationship between orientation selectivity and elongation of population units within the visual cortex.

## 1 INTRODUCTION

The region of visual space eliciting the response of a particular neuron in the visual cortex determinates the receptive field for that neuron (Felleman & Van Essen, 1987; Hubel & Wiesel, 1962). The topographical structure of the visual system can be extended in that the concept of receptive fields also applies to larger populations of neurons as population receptive field (pRF) (Engel, 1997; Victor, Purpura, Katz, & Mao, 1994). This pRF for a larger group of neurons shows similar characteristics as the fields of the single units forming that group (Wandell, 1999). Functional magnetic resonance imaging was employed to quantify spatial parameters of the pRFs, by eliciting a wave of visual excitation along the visual cortex (Engel, 1997; Engel et al., 1994). Using a variety of stimulation protocols (Dumoulin & Wandell, 2008; Greene, Dumoulin, Harvey, & Ress, 2014; Sereno et al., 1995) and estimation methods (direct fit, topographic fit), the spatial pRF properties such as of location and size (Amano, Wandell, & Dumoulin, 2009; Zuiderbaan, Harvey, & Dumoulin, 2012) could be estimated throughout the visual system with a high degree of intra‐subject reliability (van Dijk, de Haas, Moutsiana, & Schwarzkopf, 2016).

To create a less artificial representation of the spatial extend of the pRF, Merkel et al. (Merkel, Hopf, & Schoenfeld, 2018) used a back‐projection method to quantify the spatial elongation and orientation of the receptive fields. The observed distribution of these elongation parameters throughout the visual system suggests a stronger elongation within the upper visual field that increases with higher eccentricities. Furthermore, the elongated pRFs of all four quadrants show a clear radial bias, that is, they are oriented toward the fovea. These results are also supported by estimations of spatial elongation parameters using a non‐isotropic gaussian model (Silson, Reynolds, Kravitz, & Baker, 2018). Single‐cell studies suggest that the collinearity between the orientation of a receptive field and its axial position is a result of laterally connected orientation columns along regions of similar polar angles (Bosking, Zhang, Schofield, & Fitzpatrick, 1997; Chapman, Zahs, & Stryker, 1991; Ts'o, Gilbert, & Wiesel, 1986). Evidence for the close relationship between orientation selectivity of populations of neurons and their axial position was provided before (Freeman, Brouwer, Heeger, & Merriam, 2011). Merkel et al., however, showed that this also applies to the orientation of elongation of pRFs and axial position (Merkel et al., 2018). Elongation and orientation tuning are highly correlated (Anzai, Ohzawa, & Freeman, 1999; Jones, Stepnoski, & Palmer, 1987).

The current study aims to link orientation selectivity directly to the elongation of the receptive field at the population level by globally manipulating the orientation bias while measuring spatial pRF characteristics. The hypothesis is that changing orientation tuning of neural populations is altering pRF elongations particularly at axial positions parallel and orthogonal to the affected orientation.

Since scanning pRFs with fMRI is time‐consuming, the modulation of orientation selectivity must be characterized by a fairly time constant process. For this purpose, adaptation was employed as a low‐level manipulation to globally decrease the responsiveness toward one particular orientation. Since the response saturation of the visual system toward the adapter orientation follows approximately a power function (Dong, Engel, & Bao, 2014; Greenlee, Georgeson, Magnussen, & Harris, 1991), a stable modulation of orientation selectivity can be achieved in a fraction of the duration needed for the spatial sampling of the receptive fields. Previous studies showed that adaptation‐related activity modulations remain stable over prolonged exposure times (>1 hr)(Bao & Engel, 2012; Magnussen & Greenlee, 1985), and thus would be ideal to use during receptive field measurements.

**Figure 1:**
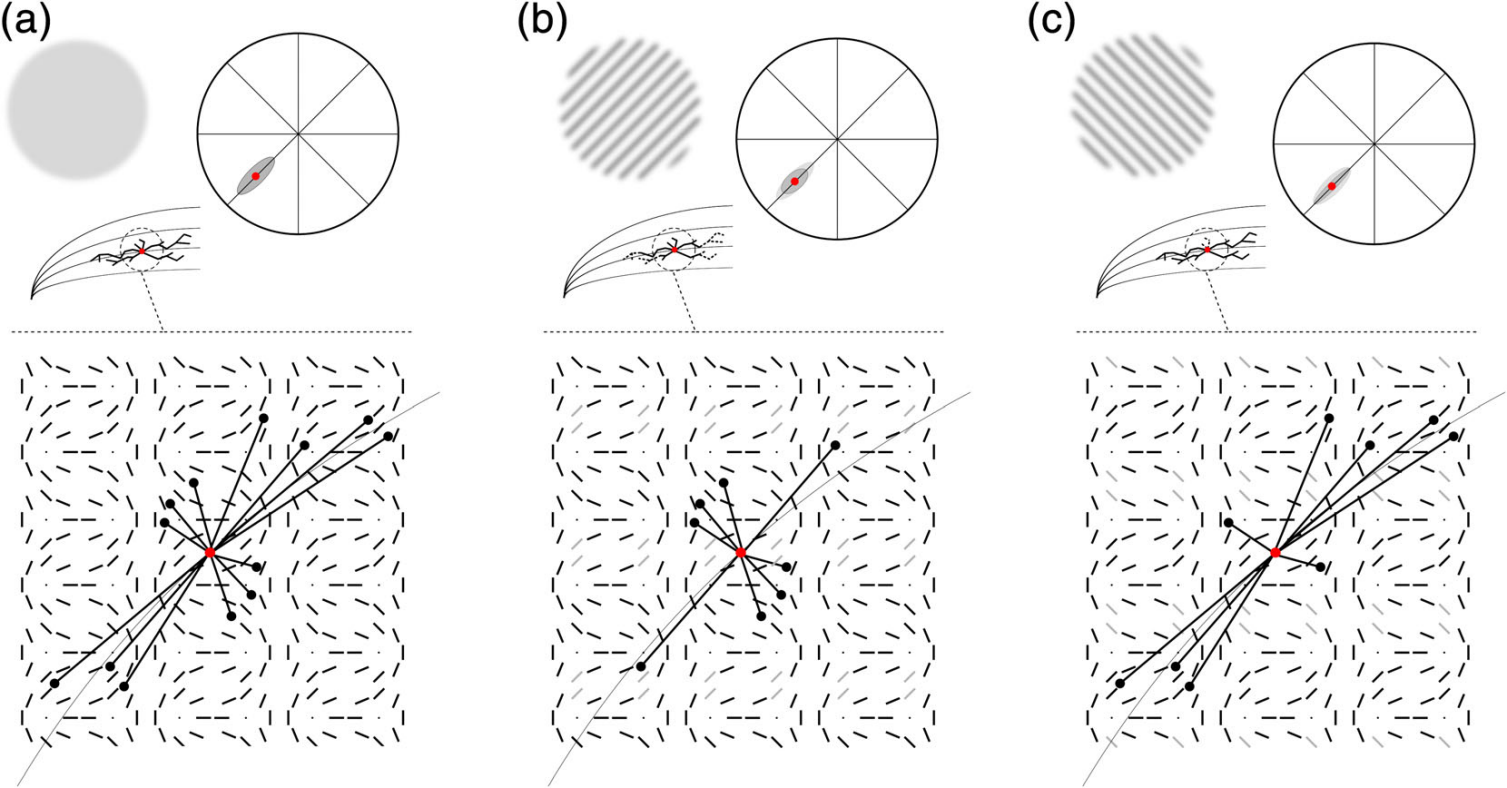
(a) Horizontal connections within a patch of orientation columns in the early visual cortex form receptive field elongations. Those connections stretch mostly along axial topographical directions, connecting units with parallel orientations, therefore creating a radial bias for the direction of receptive field elongations. The model is shown exemplary for a unit (in red) within the lower left visual field at an axial position of 225°. (b) Hypothesized effect of a continuous orientation adapter on this network. At polar positions parallel to the adapter orientation a reduction of elongation should occur due to a diminished output of adjacent units of the same orientation. (c) During the presentation of an orthogonal orientation adapter however, the same units should on the other hand exhibit a stronger elongation since parallel contributions remain largely unaffected while orthogonal connections are reduced

Assuming a strong link between orientation selectivity and spatial RF elongation, reducing the responsiveness of the visual system toward one specific orientation we expect decreased net output of the horizontal connections at parallel axial positions, thus reducing the radial spatial bias at those positions and subsequently decreasing the spatial elongation (Figure 1b). The opposite effect should be observed at orthogonal axial positions, that is, an increase in spatial elongation combined with a higher radial bias (Figure 1c).

## 2 METHODS

### 2.1 Subjects

Five female and six male subjects participated in the experiment. All subjects gave written informed consent and had no prior history of neurological disorders. The study was approved by the local ethics board. Every participant performed in two separate sessions in order to measure their spatial pRF characteristics during continuous exposure to two differently oriented adapter stimuli. The two sessions were conducted within 1 week.

### 2.2 Stimulus

A standard sweeping bar stimulus moving across the visual field was used (Dumoulin & Wandell, 2008). The whole circular aperture had a diameter of 20° and was composed of a flickering checkerboard (8 Hz) with checks of 0.8° × 0.8° size that was revealed by a bar of 1° width moving with a speed of 0.71 deg/s. The bar drifted across the full diameter of the aperture within 28 s. Swipes were performed with varying rotations of the bar relative to the center of the aperture. Each sweep‐rotation was a 24° increment of the previous bar rotation. Each session was composed of six identical runs containing 15 swipes each, resulting in a functional scan time of app. 42 min per session.

Subjects were asked to maintain fixation on a small black dot with white surrounding in the middle of the aperture throughout the experiment. In order to facilitate fixation and ensure engagement, subjects had to detect short contrast reversals of the fixation dot (white dot with black surrounding), which could occur every 3–15 s.

Importantly, throughout the whole experiment a circular sinusoidally modulated grating subtending the full diameter of the aperture was present as a continuous adapter. The two sessions differed solely in the orientation of the adapter of either 45° or 135°. Each subject was presented with both adapters during the two sessions in a random order. The grating had a spatial frequency of 1.5 c/deg which has been reported to exhibit stable BOLD adaptation effects throughout the visual cortex (Larsson, Landy, & Heeger, 2006) and a low Michelson contrast of 2.4%.

### 2.3 fMRI

Scans were performed using a 3T Siemens Prisma scanner. In each session six runs of 220 functional scans each were collected using a T2 echo‐planar pulse sequence (2.0 × 2.0 × 2.0 mm, TR = 2000 ms, TE = 30 ms, FA = 90°, FOV = 256 mm). Slice acquisition was aligned perpendicular to the calcarine fissure covering the occipital lobe with 28 slices. In the beginning of the first session, a structural MPRAGE image was acquired (1.0 × 1.0 × 1.0 mm, TR = 2,500 ms, FA = 0°, FOV = 256 mm) for each subject for subsequent cortical surface reconstruction using freesurfer (Dale, Fischl, & Sereno, 1999; Fischl, Liu, & Dale, 2001).

### 2.4 Analysis

The spatial characteristics of pRFs within the visual cortex were compared between the two sessions for each subject, examining the effects of adaptation to orientation on spatial pRF features.

Functional images of each run were preprocessed using SPM12 (http://www.fil.ion.ucl.ac.uk/spm) for each session. The preprocessing step included slice time correction to the first slice of each scan, spatial realignment to each scan of each run and to the first scan of each session and a spatial smoothing with a gaussian filter with a 2 × 2 × 2 mm FWHM kernel.

The detailed pRF back projection procedure used to calculate the 2D spatial receptive field profile for each voxels time‐series is described in Merkel et al. (Merkel et al., 2018). In short, the BOLD signals captured for each swipe direction have been detrended, z‐scored, and deconvolved with the standard SPM hemodynamic response function. Each of those resulting BOLD functions was subsequently projected onto the 2D stimulus space along their respective swipe rotation and integrated into an overall 2D signal space. Thus, a spatial representation of a receptive field profile for each voxel is obtained.

RF characteristics are measured within those representations by first defining the center of the receptive field as the location of maximum signal within the profile, thus deriving eccentricity and polar angle. The mean integrated signal intensity as a function of radius around the maximum of the profile was used to measure receptive field size. Hereby the first minimum of the derivative of the integrated signal indicated the radius of the receptive field. Next, the orientation of the RF profile was deduced from the maximum mean signal along each of the 15 swipe directions within the previously defined circular receptive field region. The elongation index was defined as the ratio of that maximum mean signal along the orientation of the field and the mean signal along the orthogonal orientation within the same circular region, with values ranging from 0 to 1. Note, that smaller values for the elongation index therefore indicate an elevated ellipticity.

Each of the RF characteristics was mapped from the voxel space onto the individuals' cortical surface space using their structural surface reconstruction. Using eccentricity and polar angle maps, ventral and dorsal early visual areas V1, V2, and V3 for the left and right hemispheres were manually delineated within the surface space for each subject. Vertex values for each of the visual areas were projected onto a separate visual field space, referencing the stimulus aperture, based on their location parameters. Thus, the distribution of the spatial receptive field parameters throughout the visual field could be examined within a universal reference space (the stimulus aperture) across all subjects and sessions irrespective of differences in size and shape of visual cortical surfaces.

Next, the effect of the continuous adapter stimulus on well‐established spatial RF parameter distributions as determined by previous RF back‐projection paradigms was examined. Therefore, a two‐factorial rANOVA with factors adapter session (45°/135°) and visual area (V1/V2/V3) was used to carve out any systematic differences in receptive field sizes. Similarly, to confirm differences in the degree of elongation between the upper and lower visual field a two‐factorial rANOVA for each visual area was performed across adapter sessions (45°/135°) and visual fields (upper/lower). The degree of collinearity between the receptive field's orientation of elongation and its axial position reflecting the global radial bias, was calculated for each session and visual area using the circular correlation coefficient (Fisher & Lee, 1983).

Preliminary data exhibited specific modulatory effects of orientation selectivity between hemifields on spatial receptive field characteristics exclusively within the lower visual field. Therefore, subsequent statistical tests were performed on data from the lower visual quadrants only. The ellipticity indexes across the lower visual field was subject to considerable random intersubject as well as intersession variation between measurements and was therefore z‐standardized for each session across the entire visual field and visual hierarchy. Collinearity data were calculated for the left and right lower visual field by correlating orientation and axial position of all pRFs within each of the two quadrants separately for each session of each subject using the circular correlation coefficient.

In order to determine whether a continuous modulation of orientation selectivity throughout the visual cortex, changes the elevation and radial bias of RF elongations between different quadrants of the visual field as predicted, a three‐factorial repeated measures ANOVA was performed to examine differences in elongation and collinearity between adapter sessions (45°/135°), hemispheres (left/right) and visual areas (V1/V2/V3). Furthermore, ellipticity and collinearity data were collapsed across the visual hierarchy and tested for differences between sessions (45°/135°) and hemispheres (left/right) using additional post‐hoc paired‐t‐tests. Investigating potential differences of individual visual areas in the contribution of modulatory influences of spatial receptive field characteristics, changes in elongation and radial bias were tested between sessions (45°/135°) and hemispheres (left/right) in each visual area separately as well using additional two‐factorial rANOVAs.

In order to identify axial positions exhibiting the strongest modulatory effects, the z‐standardized elongation data were averaged into 10° bins from 180° to 360° and compared using post hoc paired *t* tests between the two sessions.

For any of the aforementioned repeated measures ANOVAs containing more than two levels, Greenhouse–Geisser correction was applied.

## 3 RESULTS

**Figure 2:**
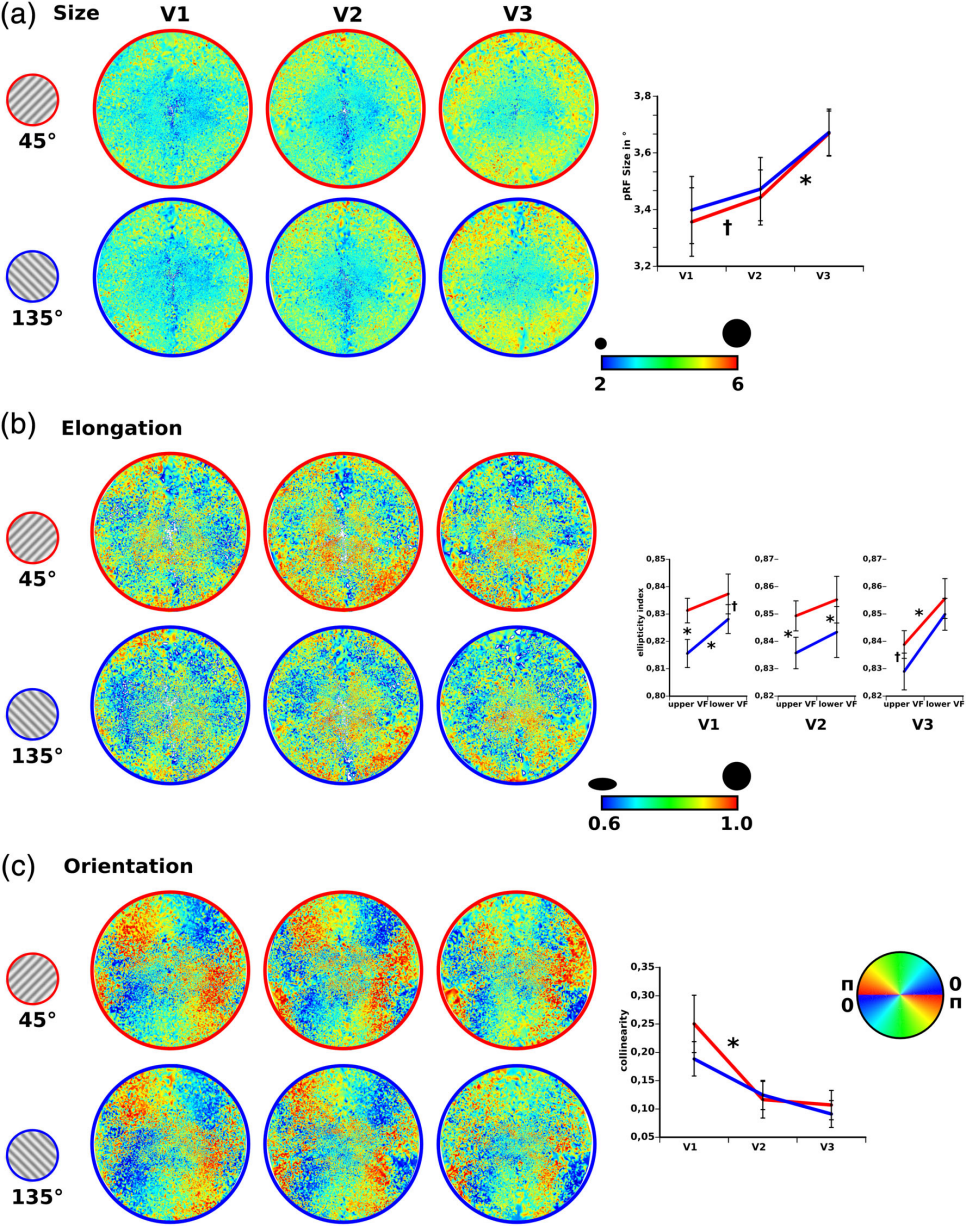
Spatial population receptive field characteristics projected onto the visual field space. For illustrative purposes only, receptive field properties were approximated across the visual field using a Delaunay triangulation of all receptive field locations. The resulting tessellation was additionally spatially smoothed. (a) Receptive field size increased along the visual hierarchy. The orientation adapter did not show any effect on the course of receptive field size change. (b) Replicating previous reports, receptive field elongation was higher within the upper visual field (smaller elongation parameter). During presentation of the 45° orientation adapter generally less elongation was observed. (c) Significant collinearities between the polar position of the receptive fields and their direction of elongations were found. Those collinearities decreased along the visual hierarchy. The orientation adapter did not show any differential effects on the collinearities

In line with previous results we observed increasing receptive field sizes along the visual hierarchy (F[2,20] = 21.86; *p* < .001), with V3 showing larger fields than V2 (t[10] = 6.03, *p* < .001) and V2 in turn exhibiting larger field sizes than V1 (t[10] = 2.14, *p* < .059) (Figure 2a). This field size effect did not show any modulation by the continuous presentation of different adapters during receptive field scanning (F[1,10] = 0.59; *p* > .46). Furthermore, there was no general receptive field size difference between adapter sessions (F[2,20] = 0.3; *p* > .64).

Regarding the spatial shape of the receptive fields, we observed a stronger elongation within the upper compared to the lower visual fields for V1 (F[1,10] = 7.21; *p* < .023) and V3 (F[1,10] = 6.1, *p* < .033) but not for V2 (F[1,10] = 0.41, *p* > .53). This also replicates previous findings (Figure 2b). Interestingly however, a significant decrease of elongation for both visual fields could be observed during the 45° adapter session in V1 (F[1,10] = 10.23, *p* < .01) and V2 (F[1,10] = 14.85, *p* < .03) with V3 failing to show a similar effect (F[1,10] = 3.08, *p* = .11). No interaction effects between adapter session and upper/lower visual field difference were found for the elongation parameter in any visual area (V1: F[1,10] = 2.82, *p* > .12, V2: F(1,10) = 0.25, *p* > .63, V3: F[1,10] = 0.78, *p* > .39).

The radial bias across the visual field operationalized as the collinearity between the axial positions of the receptive fields and their orientation of elongation did exhibit a decrease across the visual hierarchy (F[2,20] = 12.24; *p* < .001) with a mean circular correlation coefficient of 0.22 for V1 being significantly higher than 0.12 for V2 (t[10] = 4.73; *p* < .001) and 0.10 for V3 (t[10] = 4.02; *p* < .02) (Figure 2c). In general, collinearity across the whole visual field was not modulated by the different orientation adapters (F[1,10] = 0.72; *p* > .42).

**Figure 3:**
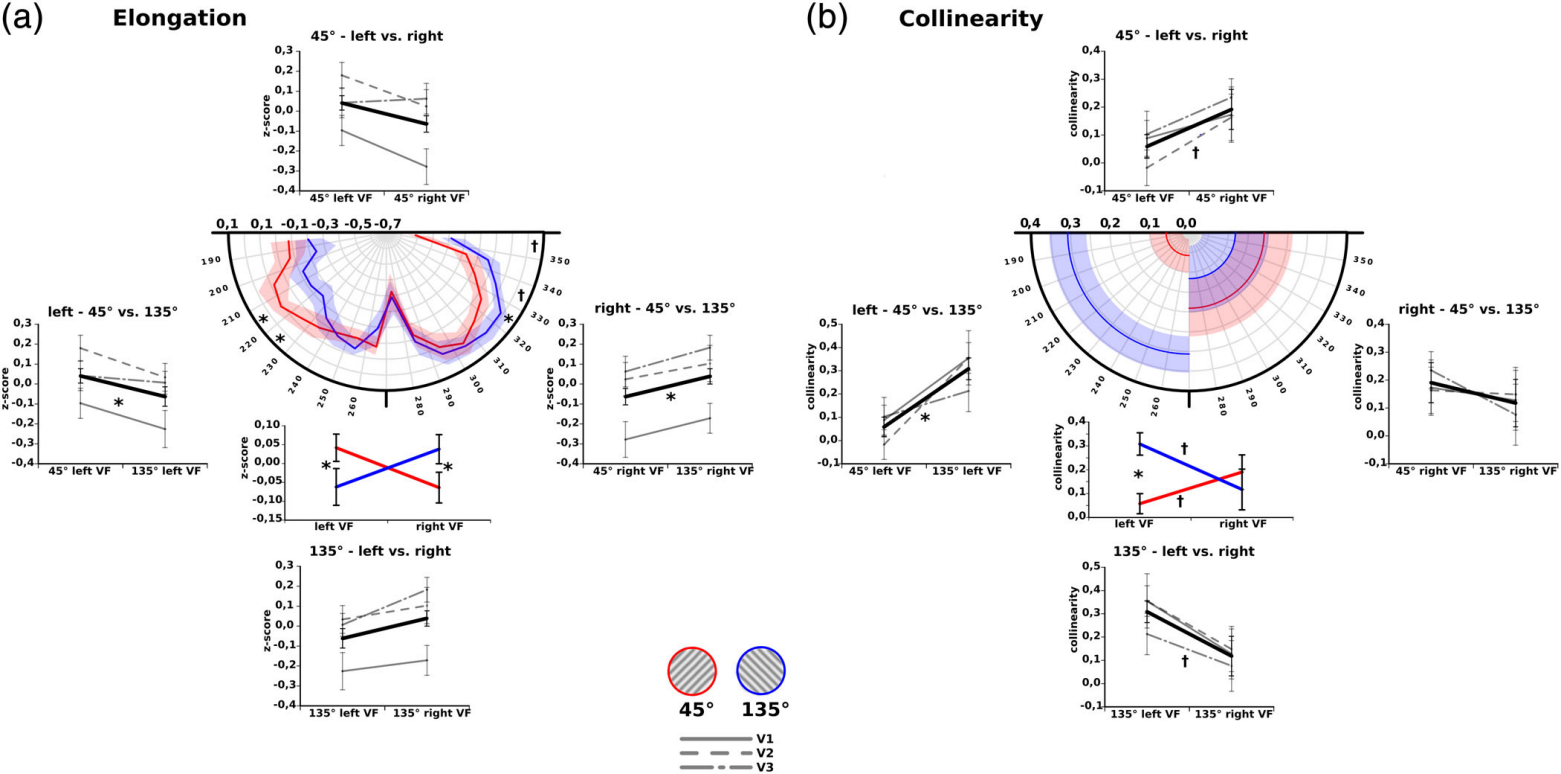
Interaction effects of receptive field parameters of elongation and ellipticity between hemisphere and adapter collapsed across the visual hierarchy (a) Differences in elongation were identified between adapter sessions within the lower left and lower right quadrant. Within the lower left hemifield receptive fields turned out to be more elongated during the 135° session than during the 45° adapter session. Stronger elongations within the right hemifield were found during the 45° session compared to the 135° session. These effects were strongest at oblique polar positions. (b) Mirroring elongation results, receptive fields were more radially oriented within the lower left quadrant during the 135° session relative to the 45° session. Moreover, the direction of differences in collinearity between left and right lower quadrants reversed for the different adapter sessions

Next, in order to investigate potential changes in the spatial characteristics of the pRFs induced by different adapter stimuli, differences in the elongation indices of vertices between sessions (45°/135°), hemispheres (left/right), and visual fields (V2/V2/V3) were tested. Importantly, the test revealed a significant interaction between adapter session and hemisphere (F[1,10] = 6.43, *p* < .03). Moreover, the ellipticity index did not show a main effect for session (F[1,10] = 0.15, *p* > .9) or hemisphere (F[1,10] = 0.002, *p* > .9). However, the elongation index did show changes across the visual hierarchy (F[2,20] = 5.78, *p* < .015). The crucial interaction effect was mainly driven by more elongated fields within the lower left quadrant during the 135°‐session compared to the 45°‐session (t[10] = 2.48, *p* < .033) and the adverse effect of more elongated fields within the lower right quadrant during the 45°‐session compared to the 135°‐session (t[10] = −2.5, *p* < .031) (Figure 3a).

Interestingly, differences in the degree of collinearity between the orientations of the receptive fields and their axial position within the left and right lower visual field during both adapter sessions were in line with the results of the elongation measure. In general, pRFs within lower visual field quadrants exhibiting higher elongations also showed an increased radial bias (Figure 3a,b). Changes in collinearity between sessions, hemispheres and visual hierarchy revealed a significant interaction between session and hemisphere (F[1,10] = 5.181, *p* < .046) (Figure 3b). Differences in the collinearity within the lower quadrants between the adapter sessions were observed due to a higher radial bias within the lower left quadrant during the 135°‐session compared to the 45°‐session (t[10] = 4.94, *p* < .001) and a trend toward higher radially oriented receptive fields within the left compared to the right quadrant during the 45°‐session (t[10] = −1.94, *p* > .081). In contrast, during the 135°‐session receptive fields trended to exhibit higher radial biases within the lower right quadrant compared to the lower left quadrant (t[10] = 2.03, *p* > .07).

**Figure 4:**
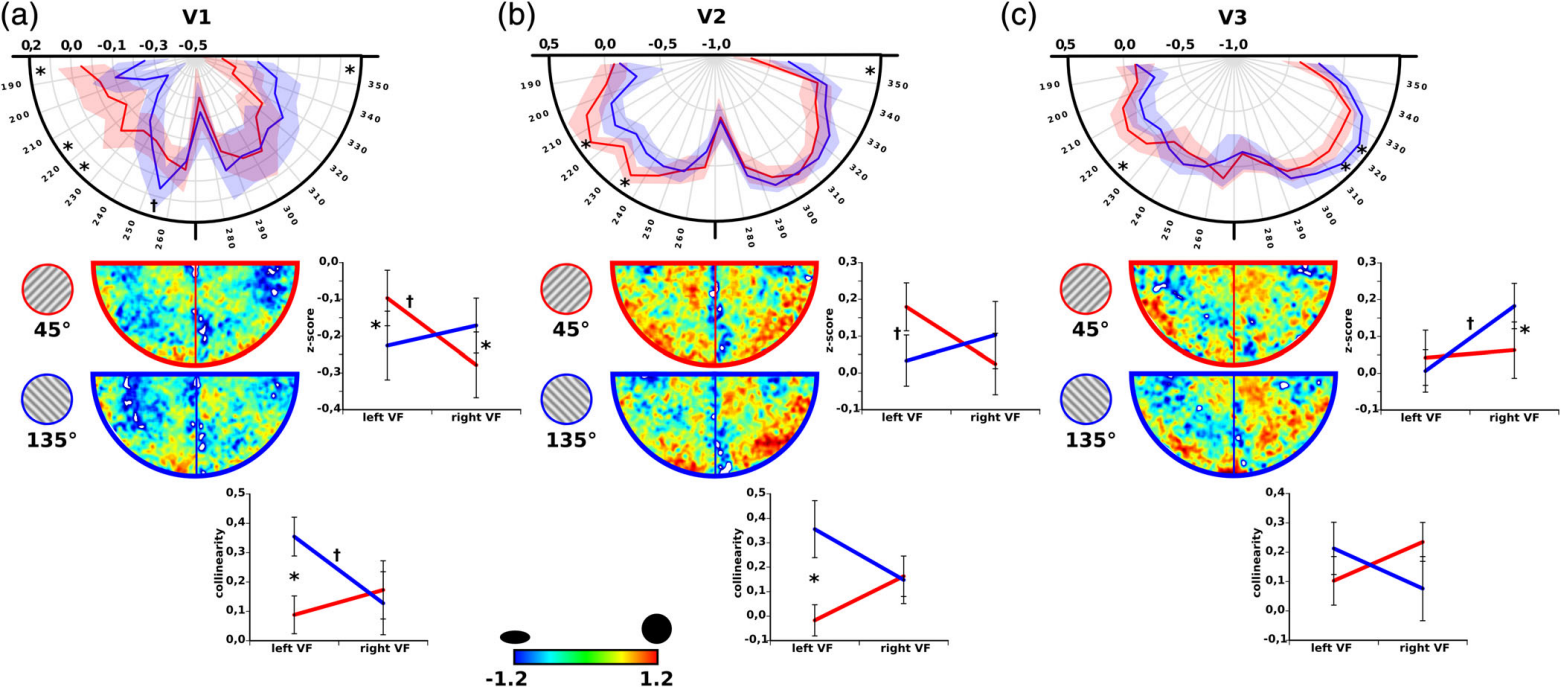
Z‐scored spatial population receptive field elongation projected onto the visual field space for each of the visual areas separately. The largest effects for field elongation differences between adapter sessions is observable within the polar plots for each visual area. Note that the interaction plots for the elongation parameter and the collinearity parameter closely resemble each other

Subsequently, the interaction effects for elongation and collinearity between adapter sessions and lower visual quadrants were tested separately for each of the visual areas using two‐factorial repeated measures analysis of variance (rANOVAs) (Figure 4). Within V1 receptive field elongation showed a significant interaction between lower quadrants and sessions (F[1,10] = 6.49, *p* < .029) while the radial bias exhibited a similar trend (F[1,10] = 3.75, *p* > .082). Both parameters indicated a higher elongation oriented toward the fovea for receptive fields within the lower left quadrant during the 135°‐session (elongation: t[10] = 2.78, *p* < .019; collinearity: t[10] = −3.08, *p* < .012) while the elongation parameter showed the adverse relationship during the 45°‐session (t[10] = −2.52, *p* < .031) (Figure 4a). The interaction effect between session and hemisphere showed trends toward the same direction for ellipticity (F[1,10] = 3.71, *p* > .083) and collinearity (F[1,10] = 3.57, *p* > .088) within area V2. These effects originated in differences for the parameters within the lower left quadrant between adapter sessions similar to area V1 (elongation: t[10] = 1.89, *p* > .089; collinearity: t[10] = −2.82, *p* < .018) (Figure 4b). While interaction effects failed to become significant within area V3 (elongation: F[1,10] = 2.78, *p* > .126; collinearity: F[1,10] = 2.342, *p* > .157), receptive fields within the lower right quadrant exhibited larger elongation during the 45°‐session compared to the 135°‐session (t[10] = −2.49, *p* < .032) (Figure 4c).

Individual elongations were averaged into 10° bins of polar angle from 180° to 360° and subsequently compared between adapter sessions in order to locate the axial position at which spatial elongation of pRFs showed the highest degree of modulation between adapter sessions. Figure 3a displays the peak of elongation differences between sessions mostly at oblique positions around 210° to 230° within the lower left quadrant and 320° to 330° within the lower right quadrant.

## 4 DISCUSSION

The current results suggest that adaptation to an orientation modulates the spatial extent of pRFs in the visual cortex. More specifically, pRFs exhibit larger elongations with increased radial bias at axial locations orthogonal to an orientation of a simultaneously presented adapter stimulus compared to parallel locations, at which receptive fields spatial elongations and orientation biases decrease.

These results indicate a causal functional relationship between orientation tuning, axial position and receptive field shape of measured population units within the visual cortex. An indirect association has been suggested before, by showing a high degree of radial bias for orientation selectivity within the visual cortex, thus a correlation with the axial position of population units (Freeman et al., 2011; Sasaki et al., 2006). Furthermore, the cells orientation preference in the early visual cortex was shown to be parallel to its long axis (Hubel & Wiesel, 1962; Reid & Alonso, 1995). A general association between orientation selectivity and receptive fields elongation could also be observed previously (Anzai et al., 1999; Jones et al., 1987). Alexander et al. (Alexander, Bourke, Sheridan, Konstandatos, & Wright, 2004; Alexander & Van Leeuwen, 2010) described an architectural model of horizontal visual cortex connections that may account for the aforementioned associations. Orientation specific lateral connections (Gilbert & Wiesel, 1989; Ts'o et al., 1986) are associated with an elongation along the axis parallel to the orientation (Fitzpatrick, 1996). Importantly, this axis corresponds to the underlying global polar position of that unit (Bosking et al., 1997; Chapman et al., 1991). The current results confirm this causal relationship by showing that the spatial field elongation and orientation of the unit is directly modulated by changing its orientation selectivity. Hereby, the global adaptation to a specific orientation is likely to reduce the net output of parallelly oriented contributions of units at corresponding axial positions, hence, reducing the unit's elongation and consequently its radial orientation.

Although recently some studies investigated the modulatory effect of visual attention on spatial pRF properties like location and size, changes in field elongation and field orientation have never been described before. Most approaches investigated the top–down modulation of spatial visual attention modeled as an attentional gain field (Kay, Weiner, & Grill‐Spector, 2015; Klein, Harvey, & Dumoulin, 2014). The observations were changes in receptive field positions toward the attended location (Klein et al., 2014; Vo, Sprague, & Serences, 2017) as well as an increase in pRF size (Kay et al., 2015; Sheremata & Silver, 2015; van Es, Theeuwes, & Knapen, 2018). However, these changes were absent in early visual areas (Kay et al., 2015; Vo et al., 2017).

In the current study, we employed the low‐level function of adaptation to adjust the visual systems orientation tuning. Adaptation produces robust effects that are detached from top–down modulations (Moradi & Shimojo, 2004). Moreover, adaptation to orientation strongly modulates early visual cortex activity, reducing the BOLD signal after prolonged exposure to an orientation stimulus (S. A. Engel, 2005; Larsson et al., 2006) in this region. With this approach we found receptive field propriety changes in early visual areas.

However, some aspects of the current results regarding the suggested general link between orientation tuning and receptive field elongation across the visual field need further attention. Interestingly, the modulatory effect could only be observed within the lower visual field. This might suggest that the orientation adapter mainly affects less oriented population units, since receptive fields in the upper visual field have a less isomorphic shape than within the lower visual field (Merkel et al., 2018; Silson et al., 2018). Therefore, adaptation to an orientation would be expected to primarily increase spatial elongations by decreasing orthogonal contributions instead of diminishing parallel local network connections. Importantly, the current data set shows relatively small modulatory effects of global orientation sensitivity biases on receptive field elongations. Adding faint biases to orthogonal or parallel elongation contributions for pRFs would more easily tilt isomorphic fields into one or the other direction compared to fields with biases that are already strongly established. Thus, the occurrence of adaptation effects would be more likely visible in the lower visual field.

However, a lack of modulatory effects of the orientation stimulus in the upper visual field is most likely a reflection of a more elementary perceptual lower visual field advantage of the early visual system (Fred H. Previc, 1990). A lower contrast threshold for the detection (Cameron, Talgar, & Carrasco, M., 2001; Lundh, Lennerstrand, & Derefeldt, 1983; Rijsdijk, Kroon, & van der Wildt, 1980; Maria Fatima Silva et al., 2008; Maria Fátima Silva et al., 2010) and discrimination (Cameron, Tai, & Carrasco, 2002; Carrasco, Williams, & Yeshurun, 2002) for oriented gratings presented within the lower visual field has been described. This perceptual advantage is prevalent in the discrimination of a variety of other stimulus features like luminance, color, and motion (Gibson, 1966; Levine & McAnany, 2005) as well as in a letter recognition task (Mackeben, 1999). The upper‐lower visual field asymmetry in visual acuity has been attributed to physiological differences in upper and lower field representations within the early visual system. Those differences include a higher density of ganglion cells in the retina for the lower visual field (Perry & Cowey, 1985), slightly larger LGN volume dedicated to the lower field (Connolly & Essen, 1984) and increased cortical surface of dorsal compared to ventral V1 (Tootell, Switkes, Silverman, & Hamilton, 1988; Van Essen, Newsome, & Maunsell, 1984). A combination of higher cortical magnification and smaller receptive fields within the cortical representation of the lower visual field have been associated with higher spatial resolution (He, Cavanagh, & Intriligator, 1996) and in turn a better visual acuity (Duncan & Boynton, 2003; Song, Schwarzkopf, Kanai, & Rees, 2015). pRF size asymmetries between upper and lower visual fields can be found throughout the early visual cortex (Silson et al., 2018) and are elevated at eccentricities above 2° (Maria Fatima Silva et al., 2018).

Importantly, vertical differences in the perception of oriented gratings are pronounced in a spatial frequency range that includes the 1.5 c/deg used for the current adapter stimulus (Lundh et al., 1983; Rijsdijk et al., 1980), indicating that an increased number of neural populations within the upper visual field were not sufficiently adapted during the current experiment in order to elicit a measurable difference in pRF shape. Interestingly, those same detection advantages for oriented gratings tend to be found in the left compared to the right hemifield (Christman, Kitterle, & Hellige, 1991; Maria Fatima Silva et al., 2008). This is fully consistent with the present observation of elongation and collinearity parameters being more easily modulated by the adapter stimulus within the left hemifield.

## CONFLICT OF INTERESTS

The authors have no conflicts of interest to declare.

## Data Availability

The data that support the findings of this study are available from the corresponding author upon reasonable request.
